# Diagnostic Agreement Between a General-Purpose AI Model and Retinal Specialists in Color Fundus Photography—A Pilot Study

**DOI:** 10.3390/jcm15093430

**Published:** 2026-04-30

**Authors:** Sara Vaz-Pereira, Laura Vilaverde, André Ferreira, Bernardete Pessoa

**Affiliations:** 1 Department of Ophthalmology, Faculdade de Medicina, Universidade de Lisboa, 1649-004 Lisbon, Portugal; 2 ALM Primum, 1050-078 Lisbon, Portugal; 3 Department of Ophthalmology, ULS Santo António, 4099-001 Porto, Portugal; 4 RISE-Health Research Network, Departamento de Biomedicina, Faculdade de Medicina, Universidade do Porto, Alameda Prof. Hernâni Monteiro, 4200-319 Porto, Portugal; 5 Institute for the Biomedical Sciences Abel Salazar (ICBAS), University of Porto (UMIB ICBAS-UP), 4050-346 Porto, Portugal; 6 Unit for Multidisciplinary Research in Biomedicine, Institute for the Biomedical Sciences Abel Salazar (ICBAS), University of Porto (UMIB ICBAS-UP), 4050-346 Porto, Portugal

**Keywords:** artificial intelligence, fundus photography, retinal diseases, interobserver variability, deep learning

## Abstract

**Background:** Artificial intelligence (AI) has shown strong performance in disease-specific retinal screening tasks; however, its reliability in heterogeneous clinical diagnostic settings remains unclear. This study compared a general-purpose multimodal AI model with experienced retinal specialists in the interpretation of color fundus photographs (CFPs). **Methods**: In this pilot retrospective cross-sectional study, 66 CFPs were independently evaluated by a masked retinal specialist and an AI model (Google Gemini 2.5 Flash). Diagnoses were compared with those of the unblinded treating specialist. The comparison was inherently asymmetric, as the reference specialist had access to full clinical information, whereas the masked evaluators performed image-only assessment. Agreement was assessed using weighted percent agreement and Gwet’s AC2 with quadratic weights. **Results**: Substantial agreement was observed between the two human specialists (AC2 = 0.67). In contrast, agreement between the AI model and the reference specialist was low (AC2 = −0.58). Direct comparison between the masked specialist and the AI also showed limited reliability (AC2 = −0.38). **Conclusions**: In this pilot study, the evaluated AI model demonstrated limited agreement relative to a context-informed specialist reference. These findings support cautious interpretation of consumer-facing multimodal AI in open-ended retinal image assessment and warrant validation in larger, multicenter studies.

## 1. Introduction

Retinal diseases such as diabetic retinopathy (DR), age-related macular degeneration (AMD), and inherited retinal disorders represent major causes of visual impairment and blindness worldwide [1,2]. Early detection and accurate diagnosis are critical to prevent irreversible vision loss and to guide timely therapeutic intervention. Color fundus photography remains one of the most widely used non-invasive imaging modalities for retinal evaluation in both screening programs and routine clinical practice [3].

Despite its widespread use, interpretation of color fundus photographs is inherently dependent on clinician expertise and may be subject to interobserver variability, particularly in subtle, early-stage, or overlapping pathological presentations [4,5]. These limitations, together with increasing demands for scalable screening solutions, have contributed to growing interest in automated image analysis systems designed to enhance diagnostic consistency, efficiency, and accessibility [1,6].

Artificial intelligence (AI), particularly deep learning (DL), has rapidly expanded within ophthalmology and demonstrated high diagnostic performance in retinal image analysis [6,7]. Disease-specific algorithms have achieved sensitivity and specificity comparable to retinal specialists in DR and AMD detection [8,9]. Systematic reviews and meta-analyses further confirm strong pooled diagnostic accuracy for AI-based DR detection across fundus photography and optical coherence tomography (OCT) modalities [10,11,12].

Beyond DR screening, AI applications in retinal diseases now include AMD, inherited retinal disorders, multimodal image integration, and broader retinal image interpretation [2,3,4,13]. Prospective validation studies of autonomous AI systems have demonstrated feasibility in primary care screening environments, supporting implementation under controlled conditions [14]. More recently, real-world evaluations have begun to assess performance outside curated datasets [15]. Recent studies have also explored the use of retinal imaging as a biomarker for systemic diseases, including neurologic and renal conditions, further expanding the scope of AI applications in ophthalmology [16,17,18,19].

However, comparative analyses between AI systems and clinicians indicate that reported equivalence often depends on predefined endpoints, curated datasets, and retrospective validation frameworks [5,18,19]. Methodological heterogeneity, dataset bias, limited external validation, and reporting inconsistencies remain important concerns [5,18]. Moreover, translational challenges—including generalizability, clinical workflow integration, and regulatory oversight—continue to limit safe and scalable deployment [2,18].

Recent advances in multimodal AI have led to the widespread availability of general-purpose platforms capable of processing medical images. Unlike disease-specific medical algorithms, these publicly accessible systems are not developed or regulated as medical diagnostic devices [1,5]. Nevertheless, patients increasingly have direct access to their retinal images and may independently upload them to widely available AI platforms outside regulated clinical environments [5]. The diagnostic reliability of such non-specialized AI models in heterogeneous routine retinal scenarios therefore remains insufficiently characterized.

The present study aims to evaluate the diagnostic agreement between a general-purpose multimodal AI model and experienced retinal specialists in the interpretation of diverse color fundus photographs obtained in routine clinical practice.

## 2. Materials and Methods

This pilot retrospective cross-sectional study included consecutive color fundus photographs (CFPs) obtained between January and March 2025 from patients under the care of a single retinal specialist (SVP). This study adhered to the tenets of the Declaration of Helsinki. In accordance with local institutional policies governing retrospective studies based exclusively on anonymized clinical data, formal ethics committee review and approval were not required, and the requirement for informed consent was waived.

Color fundus photographs were acquired during routine clinical assessment using the iCare EIDON widefield TrueColor Confocal fundus imaging system. Consecutive color fundus photographs were included without diagnostic preselection. One image per eye was included in the analysis. In cases where both eyes from the same patient were included, potential inter-eye correlation was not accounted for. Images were not excluded based on the presence of mixed or multiple retinal pathologies. Only images considered non-diagnostic due to insufficient image quality were excluded from the analysis. Demographic and clinical information were recorded contemporaneously by the treating physician at the time of image acquisition.

Each CFP was independently evaluated by two masked assessors: a senior retinal specialist (BP) and a multimodal general-purpose artificial intelligence system (Google Gemini 2.5 Flash) capable of image-based inference. The AI model (Google Gemini 2.5 Flash with Deep Research capabilities) was accessed via a web-based interface on 12 October 2025, using a MacBook Air (Apple M4 chip, Apple Inc., Cupertino, CA, USA) running macOS Sequoia 15.5 and Google Chrome. All images were anonymized prior to analysis. Given the nature of publicly accessible multimodal AI systems, no control over model versioning, determinism, or response variability was possible. The AI system was prompted with a single open-ended question asking for the most likely diagnosis based solely on the color fundus photograph, without a constrained label set or additional clinical metadata. This approach was chosen to mirror the human grading task, as human graders were also not provided with a predefined list of diagnostic labels or supplementary clinical information. All AI responses were recorded *verbatim*. For agreement analysis, the raw outputs were mapped to the study disease categories used in the manuscript (including AMD, DR, RVO, and other retinal diseases). Synonymous or closely related diagnostic terms were normalized at the disease-group level. This mapping was performed by SVP according to prespecified diagnostic grouping criteria. In cases where the specific diagnosis did not correspond to one of the main study categories, the output was classified as “other.” No materially ambiguous or true multi-label outputs were encountered. The standardized prompt and representative AI outputs, together with their corresponding mapped diagnoses, are provided in Appendix A (Table A1). A supplementary decision table illustrating representative examples of agreement, partial agreement, and disagreement classifications is also provided in Appendix A (Table A2). Diagnostic outputs from both evaluators were compared with the reference diagnosis established by the unblinded treating retinal specialist (SVP), who had access to the complete clinical context and ancillary diagnostic data.

This design introduces an inherent asymmetry, as the masked retinal specialist and the AI system were restricted to image-only interpretation, whereas the reference diagnosis reflects clinically integrated decision-making. Therefore, the comparison should be interpreted as an evaluation of agreement under constrained image-based conditions rather than a direct assessment of diagnostic accuracy.

Agreement was categorized using a three-level ordinal scale: agreement, partial agreement, and disagreement. Partial agreement was defined according to prespecified clinical criteria reflecting diagnostically related entities within the same disease spectrum. These groupings were based on standard retinal disease classifications used in routine clinical practice. For example, different stages of the same condition (e.g., early versus intermediate age-related macular degeneration) were considered partial agreement. Additionally, cases in which an evaluator correctly identified the primary diagnosis but did not capture associated, secondary, or coexisting pathological features identified by the reference specialist were also classified as partial agreement. These situations reflect incomplete characterization of complex or multimorbid retinal conditions rather than entirely incorrect diagnoses.

Inter-rater agreement was quantified using both weighted percent agreement and Gwet’s agreement coefficient (AC2) with quadratic weights. Quadratic weighting was applied to account for the ordinal structure of the agreement categories and to impose progressively greater penalties for larger diagnostic discrepancies. Gwet’s AC2 was selected instead of Cohen’s kappa due to its greater robustness in situations of category prevalence imbalance and asymmetric marginal distributions, conditions under which kappa statistics may produce paradoxically low or unstable agreement estimates.

Confidence intervals for agreement coefficients were calculated at the 95% level. Agreement strength was interpreted according to the Landis and Koch benchmarks (<0.20 slight, 0.21–0.40 fair, 0.41–0.60 moderate, 0.61–0.80 substantial, >0.80 almost perfect agreement). An exploratory subgroup analysis was additionally performed according to the reference retinal diagnosis in order to evaluate agreement across different disease categories. All statistical analyses were performed using Stata version 14 (StataCorp, College Station, TX, USA), and statistical significance was defined as a two-sided *p*-value < 0.05.

## 3. Results

A total of 66 color fundus photographs from 66 eyes of 35 patients were analyzed. The mean age of the cohort was 64.8 ± 17.9 years, and 51.4% of patients were male. Demographic characteristics are summarized in Table 1.

The diagnostic distribution reflected the clinical heterogeneity typically encountered in routine retinal practice. No single pathology predominated, and the dataset included a broad spectrum of retinal conditions ranging from common disorders such as diabetic retinopathy and age-related macular degeneration to less frequent entities including retinitis pigmentosa and pachychoroid disease. Diabetic retinopathy was the most frequent diagnosis (15.2%, n = 10), followed by myopic chorioretinopathy (12.1%, n = 8). Pachychoroid disease and normal fundus findings accounted for 10.6% of cases (n = 7 each), while the “Other” category (10.6%, n = 7) comprised less frequent retinal conditions not individually represented due to small sample sizes, including posterior vitreous detachment with Weiss ring (n = 2), chorioretinal atrophy (n = 1), treated Coats disease (n = 1), retinal vascular malformation (n = 1), choroidal nevus with subretinal fluid (n = 1), and hypertensive retinopathy (n = 1). Retinitis pigmentosa represented 9.1% (n = 6). Early age-related macular degeneration and retinal vein occlusion were each observed in 7.6% of eyes (n = 5 each). Epiretinal membrane and exudative age-related macular degeneration accounted for 6.1% of cases (n = 4 each), and intermediate AMD represented 4.5% (n = 3) (Table 2). Overall, no single pathology predominated, highlighting the diverse spectrum of retinal conditions represented in the dataset.

Substantial agreement was observed between the two human specialists. The weighted percent agreement between the unblinded reference specialist (SVP) and the masked specialist (BP) was 0.80 (95% CI 0.72 to 0.87; *p* < 0.001). The corresponding Gwet’s AC2 coefficient was 0.67 (95% CI 0.52 to 0.83), indicating substantial agreement.

**Figure 1:**
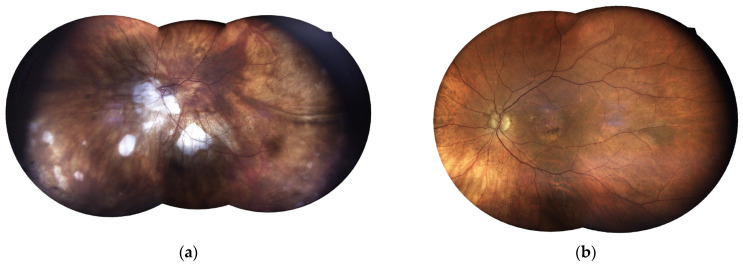
Representative examples of discordant diagnostic interpretations of color fundus photographs. (**a**) Concordant assessment between BP and SVP with the real diagnosis of patchy myopic chorioretinal atrophy, whereas the AI model (Gemini) classified the image as persistent myelinated nerve fibers; (**b**) Concordant assessment between SVP and the AI model with the real diagnosis of central serous chorioretinopathy, while BP classified the image as AMD with an atrophic component.

In contrast, agreement between the AI model and the reference specialist was markedly lower. The weighted percent agreement was 0.26 (95% CI 0.16 to 0.36; *p* < 0.001), and Gwet’s AC2 was −0.58 (95% CI −0.84 to −0.31), reflecting poor reliability after adjustment for chance agreement (Figure 1).

**Figure 2:**
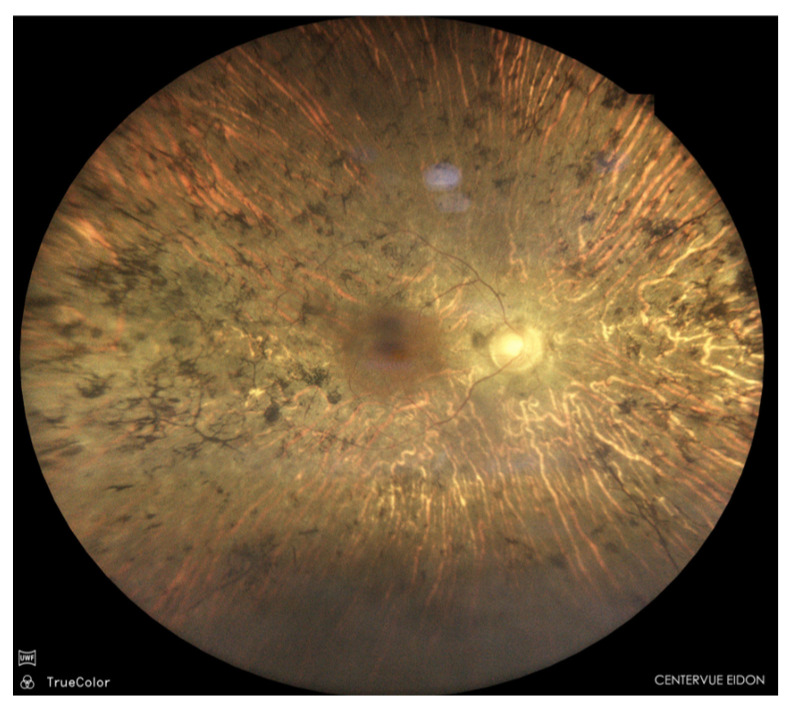
Representative example of complete diagnostic agreement among retinal specialists and the AI model in color fundus photograph interpretation. All evaluators (SVP, BP and the AI model) concurred with the reference diagnosis of retinitis pigmentosa.

Direct comparison between the masked specialist and the AI model yielded a weighted percent agreement of 0.52 (95% CI 0.41 to 0.62; *p* < 0.001) (Figure 2). The corresponding AC2 value was negative (−0.38; 95% CI −0.65 to −0.11; Table 3). Given the asymmetric category distribution and sparse subgroup counts, this chance-corrected coefficient should be interpreted cautiously. Descriptive cross-tabulation showed that exact agreement occurred in 19/66 cases (28.8%), partial agreement in 20/66 (30.3%), and disagreement in 27/66 (40.9%) (Table 4).

Collectively, these findings demonstrate a clear divergence between expert human concordance and AI-derived diagnostic outputs in this heterogeneous clinical dataset.

**Figure 3:**
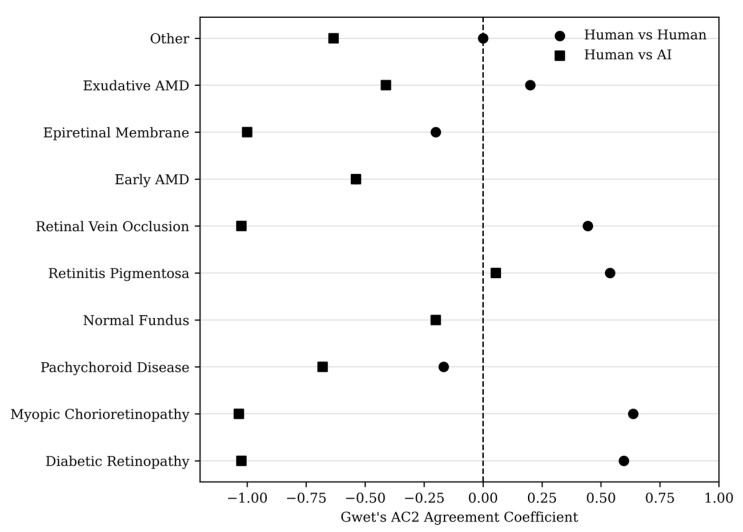
Forest-style plot showing Gwet’s AC2 agreement coefficients across retinal disease subgroups comparing human graders and the artificial intelligence model. Circles represent agreement between retinal specialists, whereas squares represent agreement between the reference specialist and the AI model. The dashed vertical line indicates the threshold of zero agreement after chance correction.

Exploratory subgroup analyses by retinal diagnosis are presented in Appendix B. Agreement between the two retinal specialists remained moderate to substantial across several diagnostic categories, particularly in conditions with distinctive fundus patterns such as myopic chorioretinopathy and retinitis pigmentosa. In contrast, agreement between the AI model and the reference specialist was generally lower. Given the small number of cases within several diagnostic categories, these subgroup findings should be interpreted as exploratory and descriptive rather than inferential, as chance-corrected agreement coefficients may be unstable in sparse multicategory settings. Accordingly, subgroup results are presented primarily as diagnosis-specific distributions, with coefficient-based estimates provided in Appendix B for transparency. The distribution of Gwet’s AC2 coefficients across diagnostic subgroups is illustrated in Figure 3.

Overall, these descriptive subgroup patterns reinforce the broader finding that agreement between the two human specialists was generally higher than agreement involving the AI model.

## 4. Discussion

In this clinically heterogeneous cohort, substantial agreement was observed between two experienced retinal specialists, supporting the internal validity of expert clinical interpretation. In contrast, the evaluated multimodal AI model demonstrated limited reliability in open-ended diagnostic assessment. This discrepancy likely reflects the fundamental difference between disease-specific medical AI systems trained on curated datasets and general-purpose multimodal AI models that lack task-specific optimization.

High diagnostic accuracy reported in prior studies has largely been derived from disease-specific algorithms optimized for predefined classification tasks, particularly in diabetic retinopathy (DR) and age-related macular degeneration (AMD) detection [6,8,9]. Multiple systematic reviews and meta-analyses confirm strong pooled performance across imaging modalities [10,11,12]. Real-world evaluations have reported encouraging results under structured deployment frameworks [13]. Additionally, pivotal prospective trials have demonstrated safe and effective implementation of autonomous AI systems in primary care screening settings [14].

However, reported equivalence between AI systems and clinicians frequently depends on curated datasets, retrospective validation frameworks, and narrowly defined diagnostic endpoints, as highlighted in systematic evaluations of AI versus clinician performance [4]. Performance may decline when models are exposed to heterogeneous data distributions or broader diagnostic categories, limiting generalizability [2,3]. Furthermore, translational barriers—including dataset representativeness, domain shift, interpretability limitations, and regulatory considerations—remain significant challenges for achieving sustained clinical impact [2,5].

Advances in multimodal AI have led to the widespread availability of general-purpose platforms capable of processing medical images, although these systems are not developed or regulated as medical diagnostic devices [1,5]. Patients increasingly have direct access to their retinal images and may independently upload them to publicly accessible AI systems. Given that such models are not specifically trained or validated for retinal disease classification, their outputs in complex and heterogeneous clinical contexts may be unreliable. The present findings therefore underscore the need for careful clinical oversight and clear differentiation between medically certified AI systems and publicly accessible general-purpose models.

While AI clearly holds promise as a screening and triage adjunct in structured environments [14], unsupervised standalone diagnostic use in heterogeneous clinical scenarios appears premature.

This study has several limitations. A key limitation is the inherent asymmetry in the reference standard. The treating retinal specialist established the reference diagnosis based on full clinical context, including access to multimodal imaging and patient information, whereas both the masked specialist and the AI model were limited to interpretation of color fundus photographs alone. As such, this study does not represent a direct like-for-like comparison of diagnostic accuracy, but rather reflects a routine clinical practice scenario in which isolated image-based interpretation is contrasted with clinically integrated diagnosis. This distinction should be considered when interpreting the lower agreement observed for the AI model, as part of this discrepancy may be attributable to the absence of clinical context rather than intrinsic model performance alone.

Additional limitations include the use of a single imaging platform, which may limit generalizability, and a sample size that may not capture the full spectrum of retinal pathology encountered in broader clinical practice. The limited sample size and the inclusion of multiple heterogeneous diagnostic categories reduce the stability of subgroup analyses and preclude definitive conclusions, supporting interpretation of this study as exploratory in nature. As both eyes from some patients were included, potential inter-eye correlation was not accounted for and may have influenced the results. An additional limitation is that the reference diagnosis and the mapping of AI outputs to diagnostic categories were performed by the same clinician (SVP). Although prespecified grouping criteria were applied, this may introduce a degree of adjudication bias. Ideally, mapping would be performed by an independent masked evaluator or through consensus review. Furthermore, the AI model was evaluated using a single standardized prompt without iterative interaction or prompt optimization, which may have influenced its performance. Importantly, this study reflects a single observed interaction with a publicly accessible AI model at a specific time point, rather than evaluation of a stable, version-controlled, and fully reproducible system.

The heterogeneous disease spectrum included in this study may better reflect routine clinical conditions than the curated datasets frequently used in AI development and validation studies. However, despite this diagnostic heterogeneity, the dataset reflects a single-center experience based on one imaging platform and one treating specialist. Therefore, external generalizability remains limited, and the findings should be interpreted within this specific clinical context. Exploratory subgroup analyses suggested variability in diagnostic agreement across retinal pathologies. However, given the small number of cases within several diagnostic categories, these findings should be interpreted as exploratory and descriptive rather than inferential, as chance-corrected multicategory agreement coefficients may be unstable in sparse datasets. Accordingly, greater emphasis is placed on the distribution of agreement, partial agreement, and disagreement, with subgroup coefficient estimates presented in Appendix B for transparency rather than as definitive comparative measures. The reduced diagnostic agreement observed in our analysis highlights the challenges faced by general-purpose AI systems when confronted with heterogeneous pathology distributions and open-ended diagnostic tasks.

From a clinical standpoint, the findings suggest that, under the specific conditions evaluated in this study, general-purpose AI systems should not be considered reliable as independent diagnostic tools for retinal disease interpretation. Nevertheless, such systems may have potential roles in preliminary triage, structured decision-support environments, or patient education when integrated within supervised clinical workflows [2,5]. Clear differentiation between medically certified diagnostic algorithms and publicly accessible AI platforms is essential to safeguard patient safety.

The increasing accessibility of multimodal AI systems capable of processing medical images raises important regulatory and ethical considerations [5]. Transparent validation frameworks, predefined intended-use specifications, and appropriate labeling of non-medical AI tools may become increasingly necessary as patients engage more directly with automated image interpretation technologies.

Future research should prioritize prospective multicenter validation of general-purpose AI systems under standardized benchmarking conditions, incorporating diverse imaging devices, heterogeneous disease spectra, and predefined diagnostic taxonomies [2,3]. Studies should also consider predefined non-inferiority margins and clinically meaningful outcome measures to better define acceptable performance thresholds. Comparative analyses between specialized disease-specific algorithms and generalist multimodal AI models may further clarify performance boundaries and appropriate clinical use cases. Additionally, investigation into hybrid human–AI collaborative models may provide a more balanced and clinically sustainable approach to AI integration in retinal care [4].

In an era of rapidly expanding public access to artificial intelligence tools, careful differentiation between validated medical AI systems and general-purpose image analysis platforms will be essential to ensure safe integration of AI into clinical ophthalmology.

## 5. Conclusions

Under the specific conditions of image-only interpretation evaluated in this pilot study, the AI model demonstrated limited agreement relative to a context-informed specialist reference. While AI technologies continue to show promise, particularly in structured screening environments, expert human oversight remains essential for safe clinical interpretation of retinal images. Continued refinement, validation, and clinically integrated deployment strategies will be critical to enhancing reliability and patient safety. These findings should be interpreted as hypothesis-generating and warrant validation in larger, multicenter studies using standardized diagnostic frameworks.

## Figures and Tables

**Table 1:** Demographic characteristics of the study population.

Characteristic	Value
Total patients, n	35
Number of eyes, n	66
Age, mean ± SD, years	64.8 ± 17.9
Male, n (%)	18 (51.4%)

**Table 2:** Diagnostic distribution of the included color fundus photographs.

Diagnosis	Cases (n)	Percent (%)	Mean Age (Years)
Diabetic Retinopathy	10	15.2	70.0 ± 7.9
Myopic Chorioretinopathy	8	12.1	71.4 ± 10.7
Pachychoroid Disease	7	10.6	69.0 ± 8.3
Normal Fundus	7	10.6	51.0 ± 16.8
Other	7	10.6	57.3 ± 22.82
Retinitis Pigmentosa	6	9.1	45.7 ± 21.9
Retinal Vein Occlusion	5	7.6	70.2 ± 10.8
Early AMD	5	7.6	65.2 ± 10.0
Epiretinal Membrane	4	6.1	75.0 ± 5.6
Exudative AMD	4	6.1	84.4 ± 1.5
Intermediate AMD	3	4.5	68.7 ± 13.3

**Table 3:** Intergrader agreement for the CFPs evaluated.

Comparison	Weighted % Agreement	Gwet’s Agreement Coefficient
SVP vs. BP	0.80	Substantial
	95% CI 0.72 to 0.87, *p* < 0.001	AC2 = 0.67, 95% CI 0.52 to 0.83
SVP vs. AI	0.26	Poor reliability
	95% CI 0.16 to 0.36, *p* < 0.001	AC2 = −0.58, 95% CI –0.84 to −0.31
BP vs. AI	0.52	Limited consistency
	95% CI 0.41 to 0.62, *p* < 0.001	AC2 = −0.38, 95% CI –0.65 to −0.11

**Table 4:** Cross-tabulation of BP and Gemini classifications relative to the 3-level agreement framework.

BP/Gemini	Disagreement	Partial Agreement	Agreement	Total
Disagreement	6 (9.1%)	0 (0.0%)	2 (3.0%)	8 (12.1%)
Partial Agreement	16 (24.2%)	5 (7.2%)	1 (1.5%)	22 (33.3%)
Agreement	25 (37.9%)	3 (4.5%)	8 (12.1%)	36 (54.5%)
Total	47 (71.2%)	8 (12.1%)	11 (16.7%)	66 (100%)

## Data Availability

The original contributions presented in this study are included in the article. Further inquiries can be directed to the corresponding author.

## Abbreviations

The following abbreviations are used in this manuscript:

AC	Agreement Coefficient
AI	Artificial Intelligence
AMD	Age-Related Macular Degeneration
BP	Bernardete Pessoa
CFPs	Color Fundus Photographs
DR	Diabetic Retinopathy
DL	Deep Learning
OCT	Optical Coherence Tomography
RVO	Retinal Vein Occlusion
SVP	Sara Vaz-Pereira

## Appendix A. AI Outputs and Diagnostic Classification Framework

This appendix provides representative examples of *verbatim* AI-generated outputs and their corresponding mapping to the predefined diagnostic categories used in the agreement analysis (Table A1). It also includes illustrative examples of how agreement, partial agreement, and disagreement classifications were assigned based on the relationship between AI outputs and the reference diagnosis (Table A2). These examples are intended to enhance transparency and are not exhaustive of all possible diagnostic combinations.

**Table A1:** Representative examples of *verbatim* AI-generated diagnostic outputs and their corresponding mapped categories.

Case	Reference Diagnosis (SVP)	AI Output (*Verbatim*)	Mapped Category
1	Retinitis Pigmentosa	Retinitis Pigmentosa	Retinitis Pigmentosa
2	Chronic Central Serous Chorioretinopathy	Chronic Central Serous Chorioretinopathy	Pachychoroid Disease
3	Myopic Chorioretinal Atrophy	Myelinated Nerve Fiber Layer	Other
4	Intermediate AMD	Early Age-Related Macular Degeneration	Early AMD
5	Pachychoroid Disease	Chronic Central Serous Chorioretinopathy	Pachychoroid Disease
6	Posterior Vitreous Detachment	Glaucoma	Other
7	Treated Coats Disease	End Stage Extensive Chorioretinal Atrophy	Other

**Table A2:** Representative examples illustrating agreement, partial agreement, and disagreement classifications between AI outputs and the reference diagnosis. These examples are illustrative and not exhaustive of all possible diagnostic combinations.

Reference Diagnosis (SVP)	AI Output	Classification
Early AMD	Intermediate AMD	Partial Agreement
Intermediate AMD	Early AMD	Partial Agreement
Chronic Central Serous Chorioretinopathy	Pachychoroid Disease	Partial Agreement
Diabetic Retinopathy	Retinal Vein Occlusion	Disagreement
Posterior Vitreous Detachment	Glaucoma	Disagreement
Retinitis Pigmentosa	Retinitis Pigmentosa	Agreement

## Appendix B. Subgroup Agreement Analysis

This appendix presents the detailed results of the exploratory subgroup analysis according to retinal diagnosis. Agreement between evaluators is summarized using both weighted percent agreement and Gwet’s AC2 coefficient for each diagnostic category (Table A3), along with the corresponding distribution of agreement, partial agreement, and disagreement (Table A4). These results are provided for transparency and should be interpreted as descriptive rather than inferential, given the small sample sizes within several diagnostic subgroups.

**Table A3:** Inter-rater agreement by diagnostic subgroup.

Diagnosis (n)	Percent Agreement (SVP vs. BP)	Gwet’s AC2	Percent Agreement (SVP vs. AI)	Gwet’s AC2	Percent Agreement (BP vs. AI)	Gwet’s AC2
Diabetic Retinopathy (10)	0.70	0.597	0.15	−1.024	0.33	−0.957
Myopic Chorioretinopathy (8)	0.78	0.636	0.09	−1.035	0.44	−0.500
Pachychoroid Disease (7)	0.54	−0.167	0.14	−0.680	0.68	0.300
Normal Fundus (7)	1.00	—	0.30	−0.200	0.30	−0.200
Retinitis Pigmentosa (6)	0.67	0.539	0.46	0.054	0.63	0.000
Retinal Vein Occlusion (5)	0.70	0.444	0.15	−1.024	0.55	−0.184
Early AMD (5)	0.20	−0.539	0.20	−0.539	—	—
Epiretinal Membrane (4)	0.44	−0.200	0.19	−1.000	0.88	0.733
Exudative AMD (4)	0.50	0.200	0.25	−0.412	0.38	−0.818
Intermediate AMD (3)	1.00	1.00	—	—	—	—
Others (7)	0.57	0.57	0.25	−0.633	0.61	0.013

(— = agreement not estimable due to perfect agreement or small sample size).

**Table A4:** Distribution of agreement categories by diagnosis for each rater.

Diagnosis (n)	BP			Gemini		
	Disagreement	Partial Agreement	Agreement	Disagreement	Partial Agreement	Agreement
Diabetic Retinopathy (10)	0 (0.0%)	3 (30.0%)	7 (70.0%)	8 (80.0%)	2 (20.0%)	0 (0.0%)
Myopic Chorioretinopathy (8)	1 (12.5%)	3 (37.5%)	4 (50.0%)	7 (87.5%)	1 (12.5%)	0 (0.0%)
Pachychoroid Disease (7)	2 (28.6%)	5 (71.4%)	0 (0.0%)	6 (85.7%)	0 (0.0%)	1 (14.3%)
Normal Fundus (7)	0 (0.0%)	0 (0.0%)	7 (100.0%)	4 (57.1%)	1 (14.3%)	2 (28.6%)
Retinitis Pigmentosa (6)	0 (0.0%)	2 (33.3%)	4 (66.7%)	3 (50.0%)	1 (16.7%)	2 (33.3%)
Retinal Vein Occlusion (5)	1 (20.0%)	2 (40.0%)	2 (40.0%)	4 (80.0%)	1 (20.0%)	0 (0.0%)
Early AMD (5)	0 (0.0%)	0 (0.0%)	5 (100.0%)	4 (80.0%)	0 (0.0%)	1 (20.0%)
Epiretinal Membrane (4)	2 (50.0%)	1 (25.0%)	1 (25.0%)	3 (75.0%)	1 (25.0%)	0 (0.0%)
Exudative AMD (4)	0 (0.0%)	2 (50.0%)	2 (50.0%)	3 (75.0%)	0 (0.0%)	1 (25.0%)
Intermediate AMD (3)	0 (0.0%)	0 (0.0%)	3 (100.0%)	0 (0.0%)	0 (0.0%)	3 (100.0%)
Others (7)	2 (28.6%)	4 (57.1%)	1 (14.3%)	5 (71.4%)	1 (14.3%)	1 (14.3%)
